# A study of tribendimidine effects *in vitro* and *in vivo* on the liver fluke *Opisthorchis felineus*

**DOI:** 10.1186/s13071-019-3288-z

**Published:** 2019-01-11

**Authors:** Maria Y. Pakharukova, Vladimir A. Samsonov, Elena A. Serbina, Viatcheslav A. Mordvinov

**Affiliations:** 1 Institute of Cytology and Genetics of the Siberian Branch of the Russian Academy of Sciences (SB RAS), 10 Lavrentiev Ave, Novosibirsk, 630090 Russia; 2 Novosibirsk State University, 2 Pirogova Str, Novosibirsk, 630090 Russia; 3 Vorozhtsov Institute of Organic Chemistry of SB RAS, Novosibirsk, Russia; 4 Institute of Systematics and Ecology of Animals, SB RAS, Novosibirsk, Russia

**Keywords:** Food-borne trematodes, *Opisthorchis felineus*, Tribendimidine, *in vivo*, *in vitro*, Hamster model

## Abstract

**Background:**

The food-borne liver fluke *Opisthorchis felineus* is an epidemiologically important species and the causative agent of opisthorchiasis across an extensive territory of Eurasia. For decades, treatment of opisthorchiasis has been based on praziquantel. Tribendimidine could be an alternative drug that has been successfully tested for *Opisthorchis viverrini* and *Clonorchis sinensis* infections. We aimed to assess tribendimidine effects in comparison with praziquantel *in vivo* and *in vitro* against the liver fluke *Opisthorchis felineus*.

**Results:**

In this study we (i) calculated half-maximal inhibitory concentrations (IC_50_) by motility tests against *O. felineus* adults and newly excysted metacercarie after tribendimidine treatment *in vitro*; (ii) determined whether tribendimidine and PZQ effects on adult liver flukes are dependent on or mediated by white blood cells; and (iii) tested *in vivo* the anthelmintic activity of tribendimidine on juvenile and adult worms. We found that the efficiency of tribendimidine *in vitro* was similar (IC_50_ = 0.23 μM for newly excysted metacercariae and 0.19 μM for adult worms) to that of praziquantel (IC_50_ 0.98 μM for newly excysted metacercariae and 0.47 μM for adult worms). The treatment of adult worms *in vivo* with praziquantel or tribendimidine at 400 mg/kg resulted in a 76% and 77.2% reduction, respectively, in the worm burden during chronic infection.

**Conclusions:**

The differences between WBR values after PZQ and TBN treatment were not significant, thus tribendimidine was as effective as praziquantel against *O. felineus* liver flukes. Given the broad-spectrum activity of tribendimidine and efficacy against *O. felineus*, this drug may be a promising candidate for the treatment of opisthorchiasis felinea and other liver fluke infections.

## Background

The liver fluke *Opisthorchis felineus* is a member of the triad of epidemiologically important species of food-borne trematodes (*O. felineus*, *O. viverrini* and *C. sinensis*), and the causative agent of opisthorchiasis felinea over an extensive territory across Eurasia [1–3]. The prevalence of *O. felineus* infection in the population of the endemic regions of Western Siberia is 10–45% [1, 2]. Human infection results from eating raw or undercooked freshwater cyprinoid fish carrying metacercariae of the parasite [1, 2]. Metacercariae excyst in the duodenum, and the juvenile parasites ascend into the bile ducts, where development into adult worms proceeds over the course of four weeks. The adult liver flukes reside within the bile ducts and gall-bladder [2] and shed eggs that enter the gastrointestinal tract and are released with feces.

The clinical manifestations and pathology induced by chronic infection with *O. felineus*, *O. viverrini* and *C. sinensis* liver flukes are similar [4, 5]. The liver flukes induce several hepatobiliary disorders, including hepatomegaly, cholangitis, periductal fibrosis, chronic inflammation, liver abscesses [2, 3, 6] and potentially cholangiocarcinoma. The International Agency for Research on Cancer classifies the flukes of the family Opisthorchiidae (*O. viverrini* and *C. sinensis*) as group 1 agents and major risk factors for cholangiocarcinoma [4].

To date, the only drug of choice for the treatment of opisthorchiasis, clonorchiasis, schistosomiasis and other fluke infections has been praziquantel (PZQ). PZQ disrupts calcium ion homeostasis and antagonizes voltage-gated calcium channels. Drug discovery for trematode infections has a high priority [7] to avoid the development of resistance to PZQ in the future. Studies on alternative drugs for the treatment of liver fluke infections have shown that tribendimidine (TBN) is a good candidate [8–10]. It is a symmetrical diamidine derivative of amidantel and was developed in China for use in humans in the mid-1980s [11]. TBN is a cholinergic agonist that is selective for nicotinic acetylcholine receptors. TBN has a broad-spectrum activity against intestinal nematodes. The efficiency of TBN against trematodes infection is variable [9, 12]. The drug is effective against *O. viverrini* and *C. sinensis* infections, but ineffective against *S. mansoni* infection [9]. To our knowledge, TBN has never been tested against *O. felineus* infection.

The aims of the present study were to (i) test *in vitro* anthelmintic activity of TBN by calculating IC_50_ values *via* motility tests on juvenile and adult *O. felineus* worms; (ii) assess if TBN and PZQ effects on liver flukes are dependent on or mediated by white blood cells; and (iii) test *in vivo* the anthelmintic activity of TBN on juvenile and adult worms.

## Methods

### Compounds

TBN [(1E,1′E)-N′,N′′-(4,4′-(1E,1′E)-(1,4-phenylenebismethan-1-yl-1-ylidene))bis(azan-1-yl-1-ylidene)bis(4,1-phenylene))bis(N,N-dimethylacetimidamide)] was synthesized and provided by the Vorozhtsov Institute of Organic Chemistry of the Siberian Branch of the Russian Academy of Sciences (Novosibirsk, Russia). Spectroscopic characteristics confirmed the structure of the compound (purity 99%).

For *in vitro* tests, PZQ (Sigma-Aldrich, St Louis, USA) and TBN were dissolved in dimethyl sulfoxide (DMSO) (Sigma-Aldrich) to obtain 1 mM stock solutions.

For *in vivo* experiments, PZQ and TBN were prepared as a suspension in an aqueous solution of 7% Tween 80 (v/v) and 3% ethanol (v/v) before oral administration (10 ml/kg) and were administered at 400 mg/kg body weight.

### Animals and infection

Syrian hamsters (*Mesocricetus auratus*) were purchased from the Animal Facility of the ICG SB RAS. Before infection, the animals were allowed to acclimate for one week at our animal facility. They were kept in groups of maximum three hamsters per cage, with free access to water and rodent food pellets. Euthanasia was performed by carbon dioxide inhalation, and every effort was made to minimize suffering. *Opisthorchis felineus* metacercariae were collected from naturally infected fish (*Leuciscus idus*) caught in the Ob River near Novosibirsk (Western Siberia) and extracted accordingly [13].

For *in vivo* experiments, 83 male Syrian hamsters (aged 6–8 weeks) were chosen randomly. The animals were orally infected with 75 *O. felineus* metacercariae.

### WBC extraction from blood

Blood samples from infected hamsters were collected by the cardiac puncture with anticoagulant (EDTA). Next, 0.3% gelatin (Sigma-Aldrich) in PBS was added to the blood samples in a 3:1 ratio, and the samples were incubated for 4–6 h at 37 °C. This step allowed the separation of blood into an upper plasma layer containing WBCs and a lower (red-blood-cell layer). The upper layer was collected and centrifuged at 2000× *g* for 5 min. The supernatant was aspirated and the pellet resuspended in PBS. This step was repeated three times. Finally, WBCs were resuspended in RPMI 1640 medium (Life Technologies, Foster City, USA) and counted.

### *In vitro* activity

Newly excysted metacercariae (NEM) were prepared from metacercariae according to a previously published protocol [13]. Adult worms were recovered from the livers of hamsters infected three months earlier and then worms were thoroughly washed with sterile saline solution (0.9% NaCl). For calculation of the half-inhibitory concentration (IC_50_), we tested the following concentrations of compounds: 0.001, 0.01, 0.1, 1, 10 and 100 μM. The DMSO concentration across different compound concentrations was 0.5% v/v. As control groups, we used flukes incubated in the medium with 0.5% DMSO. Thus, four to five adult worms or 30–40 NEM per well of a 12-well culture plate were analyzed. The worms were incubated at 37 °C for 24 h in the RPMI 1640 medium (Life Technologies) supplemented with 100 U/ml penicillin, 0.1 μg/ml streptomycin, 0.25 μg/ml amphotericin B (Sigma-Aldrich), 1% glucose and the corresponding concentration of a drug [13, 14].

After 24 h of treatment with one of the drugs, viability of the worms was evaluated under an inverted microscope (Axiovert 40CFL, Carl Zeiss, Jena, Germany) equipped with a camera (Axiocam ICC3, Zeiss) (magnification 10–50×). The experiments were repeated three times with two replicates for each concentration. The motility of viable worms was assessed on a motility scale from 0 to 3: 3, very active (similar movements as the control flukes); 2, active (reduced motility when compared to the control; however, the entire body still moving); 1, reduced viability (only movements of the oral sucker); and 0, non-motile (non-motile for 2 min) [8, 13, 14]. The IC_50_ value is defined as the concentration of a drug required to decrease the mean motility of a worm to 50% at the 24-h time point. Four-parameter logistic regression was used to calculate IC_50_ and standard error values (R package *drc 3.0-1*) [15]. The ANOVA lack-of-fit test was conducted to test the hypothesis that a proposed regression model fits the data well.

To assess the combinatorial activity of the anthelmintics and immune system, freshly extracted adult worms (3 months post-infection) were incubated overnight in the medium. After that, adult worms were incubated in the medium with TBN (0.5 μM) or PZQ (0.5 μM) and freshly extracted 4 × 10^5^ WBCs for 7 days. As control groups, we used flukes incubated in the medium with 0.5% DMSO. Mortality rates were evaluated each day. The worms were classified as dead if they had a dark color and no movement was observed for 2 min [13]. To estimate mortality rates, Kaplan-Meier survival curves were built by means of the *survival* (v.2.38) R package. Finally, statistical difference in survival log-rank (Mantel-Haenszel test) within each pair of samples was calculated.

### *In vivo* activity

The hamsters were subdivided into three batches for experimental treatment. In each batch, hamsters were infected with metacercariae extracted from the same batch of fish. In two batches, hamsters were treated 1 month post-infection (corresponding to infection with adult *O. felineus* worms) and 15 days post-infection (corresponding to infection with juvenile *O. felineus*)*.* In the remaining batch, the hamsters were treated 3 months post-infection, which corresponds to chronic infection*.*

Groups of 5–7 hamsters were treated with a single drug dose *via* oral administration. PZQ and TBN was administered at 400 mg/kg body weight. Ten days after the treatment, the hamsters were placed in new cells; whole-stool samples were collected after 3 subsequent days, and 1 g of each stool was analyzed by means of Mini Parasep concentrators (Apacor, Wokingham, UK). The number of eggs per gram of stool (EPG) was determined. Egg reduction rates (ERR) were calculated as follows: (1 – Arithmetic Mean EPG at follow-up)/Arithmetic Mean EPG in control × 100 [10].

Worms remaining in the hepatobiliary system on day 14 post-treatment were counted after killing the hamsters by CO_2_ asphyxiation. Drug activity was expressed as a worm burden reduction (WBR) as described elsewhere [8, 16]. Briefly, WBRs were calculated as follows: (a − b)/a × 100, where a is the average worm count in the control group upon dissection, and b is the average worm count in the treated group upon dissection. The significance of WBR and ERR was evaluated by the Mann-Whitney U-test and Fisher’s exact test in the STATISTICA 6.0 software (Statsoft Inc, Tulsa, USA).

## Results

### *In vitro* activity

Evaluation of the compounds by standard motility tests against NEM revealed that IC_50_ of TBN (IC_50_ = 0.23 ± 0.059 μM; ANOVA lack-of-fit test: *RSS* = 0.02, *df* = 3, *P* = 0.0001) was similar to that of PZQ (IC_50_ 0.98 ± 0.18 μM; ANOVA lack-of-fit test: *RSS* = 0.008, *df* = 3, *P* = 0.02; Table 1). When the drugs were tested on adult worms, TBN effects (IC_50_ = 0.19 ± 0.1 μM; ANOVA lack-of-fit test: *RSS* = 0.006, *df* = 7, *P* = 0.0003) were also similar to that of PZQ (IC_50_ = 0.47 ± 0.05 μM; ANOVA lack-of-fit test: *RSS* = 0.009, *df* = 3, *P* = 0.05; Table 1).

**Table 1:** IC_50_ values of tribendimidine and praziquantel against newly excysted metacercariae and adult *Opisthorchis felineus* worms

Compound	NEM	Adults
Tribendimidine (TBN) (μM)	0.23 ± 0.059	0.19 ± 0.1
Praziquantel (PZQ) (μM)	0.98 ± 0.18	0.47 ± 0.05

For calculation of the half-maximal inhibitory concentration (IC_50_), we tested the following concentrations of compounds: 0.001, 0.01, 0.1, 1, 10 and 100 μM. Data are presented as IC_50_ values ± standard error (R package *drc 3.0-1*)

*Abbreviation*: NEM, newly excysted metacercariae

**Fig. 1:**
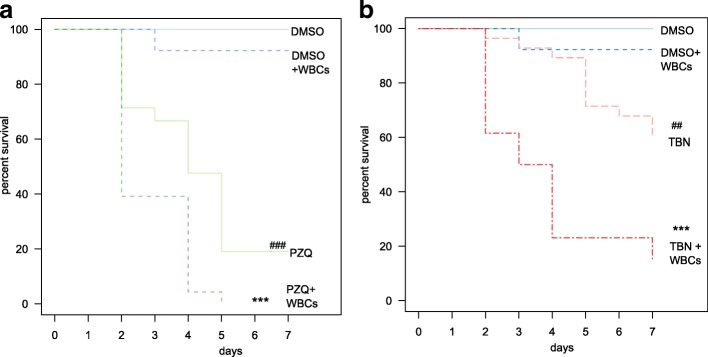
Kaplan-Meier survival curves for PZQ- (**a**) and TBN-treated (**b**) adult worms. A set of Kaplan-Meier survival curves (out of three independent experiments) is shown. *P*-values were obtained by the survival log-rank (Mantel-Haenszel) test (R package *survival* v.2.38) within each pair of samples. *A significant difference between groups (treatment *vs* treatment + WBCs), #A significant difference between groups (treatment *vs* vehicle DMSO), **^(##)^*P* < 0.01; ***^(###)^*P* < 0.001. *Abbreviations*: DMSO: dimethylsulfoxide; TBN: tribendimidine; PZQ: praziquantel; WBCs: white blood cells

The mortality of adult flukes was different between the PZQ and TBN treatment groups (Mantel-Haenszel log-rank test: *χ*^2^ = 15.8, *df* = 1, *P* < 0.0001). The mortality of adult worms in the TBN group was 40% after 7 days of treatment, as compared to 80% mortality in the PZQ group after 7 days of treatment (Fig. 1a, b).

To assess if TBN and PZQ effects on liver flukes are depending on or mediated by white blood cells, adult worms were co-cultivated in the media together with WBCs freshly extracted from the blood of the infected animals and worm mortality was assessed each following day. We found that the combined action of the drug and the immune compounds caused a much greater effect on the mortality of adult helminths. In particular, the mortality of helminths in the combined group of PZQ and WBCs significantly increased (Mantel-Haenszel log-rank test: *χ*^2^ = 4.8, *df* = 1, *P* = 0.0289; Fig. 1a) in comparison with the mortality of worms treated with PZQ (Fig. 1a). We observed a similar effect under the TBN treatment. Thus, the mortality of helminths treated with TBN and WBCs was significantly increased (Mantel-Haenszel log-rank test: *χ*^2^ = 18.7, *df* = 1, *P* < 0.0001; Fig. 1b) when compared with the mortality in the TBN group (Fig. 1b). It should be noted that the mortality of adult flukes was not different between both combined groups PZQ + WBCs and TBN + WBCs (Mantel-Haenszel log-rank test: *χ*^2^ = 2.3, *df* = 1, *P* = 0.131). WBCs had no effect on DMSO-treated helminths (Mantel-Haenszel log-rank test: *χ*^2^ = 0.9, *df* = 1, *P* = 0.337).

### *In vivo* activity

We assessed the effect of TBN at different stages of the disease, specifically 15-day and 1-month infections, which are considered acute opisthorchiasis, and 3 month chronic opisthorchiasis. The treatment of hamsters 15 days post-infection with TBN at 400 mg/kg resulted in a WBR of 90.7% (Mann-Whitney U-test: *U* = 0, *df* = 4, *P* = 0.009; Table 2). The treatment of hamsters 1 month post-infection with TBN at 400 mg/kg caused a WBR of 90% (Mann-Whitney U-test: *U* = 0, *df* = 4, *P* = 0.009; Table 2).

**Table 2:** Worm burden reduction values obtained after treatment of hamsters harboring *O. felineus* with tribendimidine or praziquantel

	Number of animals	Mean no. of worms ± SD	WBR (%)	Mean EPG ± SD	ERR (%)
Treatment of chronic infection (3 months)					
Control	13	33 ± 10	–	1863 ± 1006	–
TBN, 400 mg/kg	5	7 ± 4	76	927 ± 620	51.0
PZQ, 400 mg/kg	11	8 ± 5	77.2	nd	nd
Treatment of acute infection (1 month)					
Control^1^	5	19 ± 2	–	863 ± 279	–
Control^2^	6	34 ± 9	–	nd	–
TBN^1^, 400 mg/kg	5	2 ± 4	90	299 ± 244	65.4
PZQ^2^, 400 mg/kg	7	8 ± 6	76.5	nd	nd
Treatment of acute infection (15 days)					
TBN^1^, 400 mg/kg	5	2 ± 2	90.7	463 ± 281	46.3

Superscript numbers denote data relative to the corresponding control group

*Abbreviations*: TBN, tribendimidine; PZQ, praziquantel; SD, standard deviation, WBR, worm burden reduction rate, EPG, eggs per gram of feces, ERR, egg reduction rates, nd, not determined

Treatment with PZQ at 400 mg/kg 1 month post-infection resulted in a WBR of 76.5% (Mann-Whitney U-test: *U* = 0, *df* = 6, *P* = 0.002). According to Fisher’s exact test (*P* = 0.278; 0.447), the differences between WBR values after PZQ and TBN treatment 1 month post-infection were not significant.

Treatment of hamsters with chronic infection (3 months post-infection) with TBN at 400 mg/kg caused a WBR of 76% (Mann-Whitney U-test: *U* = 0, *df* = 4, *P* = 0.009; Table 2). For comparison, treatment of hamsters with chronic infection with PZQ resulted in a WBR of 77.2% (Mann-Whitney U-test: *U* = 0, *df* = 10, *P* = 0.014; Table 2). The differences in worm reduction rates between acute and chronic infection for TBN were not significant (Fisher’s exact test: *P* = 0.305).

## Discussion

In this study, we compared the anthelmintic activity of TBN and PZQ on the experimental model of the liver fluke infection caused by *O. felineus*. Our data indicate that the efficacy of TBN is similar to that of PZQ *in vitro* and *in vivo* on different stages of the disease: at one and three months after the infection.

TBN efficacy against *O. felineus* are in compliance with the data shown on *O. viverrini* [8–10]. The IC_50_ for TBN *in vitro* against adult *O. viverrini* was shown to be 0.05 μg/ml [8]. A similar efficacy of a single dose *in vivo* has been shown in studies against *O. viverrini*: 400 mg/kg of TBN treatment was sufficient for 95.7% WBR in hamsters four weeks after the infection [9]. High efficacy of TBN has also been demonstrated on an experimental model of clonorchiasis (4–6 weeks of *C. sinensis* infection): a 150 mg/kg dose was sufficient for 91.5% WBR, and 240 mg/kg resulted in 99.4% WBR [17].

High cure rates were observed with TBN (90%) and PZQ (90%) among *O. viverrini*-infected participants with low-intensity infections [10]; however, several studies have shown lower efficacy rates for TBN and PZQ in the treatment of moderate and heavy infections (44% for TBN and 56% for PZQ) [12, 18]. Thus, TBN has efficacy comparable to that of PZQ in the treatment of liver fluke infections. Nevertheless, TBN was shown to have a good safety profile and caused fewer adverse events [10] compared with PZQ when tested on participants with *O. viverrini* infection. In particular, in one clinical trial, participants treated with PZQ were about four times more likely to have an adverse event than those treated with TBN [10]. All adverse events in the TBN group were mild except for nausea. Moderate adverse events were reported in the PZQ group, including vertigo, nausea, fatigue, abdominal cramps and vomiting [10].

We also demonstrated that apparently there is an immunity-dependent lethal effect of TBN on the liver flukes *O. felineus*. The mortality of helminths in the combined group of TBN and WBCs significantly increased in contrast to that of worms treated with TBN alone.

Although both substances have completely different chemical structures (suggesting that they must act on different molecular targets), they have similar effects on the appearance of helminths. In particular, both drugs cause helminth immobilization and paralysis and damage the tegument [17, 19]. TBN acts as a cholinergic agonist of the B subtype and L subtype of nicotinic acetylcholine receptors [11]. In contrast, the precise mechanism of action of PZQ has not yet been elucidated (reviewed in [20]). Exposure of worms to PZQ causes a massive influx of calcium, contraction of the musculature, disruption of the tegument, and effects on calcium channels (reviewed in [20]). The disruption of the tegument presumably allows immune cells of the host to attack the parasite’s tissues and organs (reviewed in [20]). Accordingly, simultaneous administration of WBCs with PZQ in our study resulted in a higher mortality rate than that of PZQ alone (Fig. 1a). These findings are in agreement with the data on schistosomes. It has been reported that immune effector mechanisms may be synergistically involved in the action of PZQ on worms [21]. In particular, administration of rabbit anti-*Schistosoma mansoni* antisera simultaneously with PZQ yielded a greater WBR relative to PZQ alone.

The tegument of trematodes is a multinuclear syncytium that is approximately 4 μm thick and has several vital functions, including protection from immune cells of the host, absorption of nutrients, ion transport and communication with the underlying nervous system. The tegument is in direct contact with muscle fibers, ensuring an instantaneous reaction of the muscles to external stimuli, such as mechanical pressure, an ion gradient, or a gradient of nutrients [22]. Furthermore, the ability to damage the tegument has been described for other anthelmintics, such as artemether [17], artesunate [23] and mefloquine [24]. It should be noted that many anthelmintic agents act on different molecular targets but have the same effect: tegumental damage. This fact does not mean a direct effect of the drugs on a molecular target within the tegument but rather an indirect effect through disturbances in the physiology of helminths and damage to the parasite’s tissues and organs.

Mortality *in vitro* in combined groups of adult worms treated with TBN + WBCs and PZQ + WBCs was almost the same. These findings are in agreement with the results from our *in vivo* study, where respective single oral doses of TBN and PZQ resulted in similar anthelmintic effects.

## Conclusions

For decades, treatment of the liver fluke infection caused by *Opisthorchis felineus* has been based on PZQ. Our findings on an experimental model showed that TBN is also an efficient drug for therapy of this pathology. Both *in vivo* and *in vitro* experiments demonstrated its high efficacy against *O. felineus*. Hence, it can be a potential candidate for the treatment of the disease.

## Abbreviations

DMSO: Dimethylsulfoxide
EPG: Eggs per gram of feces
ERR: Egg reduction rate
IC_50_: Half-inhibitory concentration
PZQ: Praziquantel
TBN: Tribendimidine
WBCs: White blood cells
WBR: Worm burden reduction

## References

[1] Be’er SA. Biology of the agent of opisthorchiasis. Moscow: KMK Scientific Press Ltd; 2005.

[2] Pakharukova MY, Mordvinov VA (2016). The liver fluke *Opisthorchis felineus*: biology, epidemiology, and carcinogenic potential. Trans R Soc Trop Med Hyg.

[3] Pozio E, Armignacco O, Ferri F, Gomez Morales MA (2013). *Opisthorchis felineus*, an emerging infection in Italy and its implication for the European Union. Acta Trop.

[4] IARC, IARC working group on the evaluation of carcinogenic risks to humans. Biological agents. A review of human carcinogens. In: IARC Monographs on the Evaluation of Carcinogenic Risks to Humans. 2012. 100:1–441. PMC4781184 23189750

[5] Maksimova GA, Pakharukova MY, Kashina EV, Zhukova NA, Kovner AV, Lvova MN (2017). Effect of *Opisthorchis felineus* infection and dimethylnitrosamine administration on the induction of cholangiocarcinoma in Syrian hamsters. Parasitol Int.

[6] Gouveia MJ, Pakharukova MY, Laha T, Sripa B, Maksimova GA, Rinaldi G (2017). Infection with *Opisthorchis felineus* induces intraepithelial neoplasia of the biliary tract in a rodent model. Carcinogenesis.

[7] Prichard RK, Basanez MG, Boatin BA, McCarthy JS, Garcia HH, Yang GJ (2012). A research agenda for helminth diseases of humans: intervention for control and elimination. PLoS Negl Trop Dis.

[8] Keiser J, Adelfio R, Vargas M, Odermatt P, Tesana S (2013). Activity of tribendimidine and praziquantel combination therapy against the liver fluke *Opisthorchis viverrini in vitro* and *in vivo*. J Helminthol.

[9] Keiser J, Shu-Hua X, Chollet J, Tanner M, Utzinger J (2007). Evaluation of the *in vivo* activity of tribendimidine against *Schistosoma mansoni*, *Fasciola hepatica*, *Clonorchis sinensis*, and *Opisthorchis viverrini*. Antimicrob Agents Chemother.

[10] Sayasone S, Keiser J, Meister I, Vonghachack Y, Xayavong S, Senggnam K (2018). Efficacy and safety of tribendimidine *versus* praziquantel against *Opisthorchis viverrini* in Laos: an open-label, randomised, non-inferiority, phase 2 trial. Lancet Infect Dis.

[11] Robertson AP, Puttachary S, Buxton SK, Martin RJ (2015). Tribendimidine: mode of action and nAChR subtype selectivity in *Ascaris* and *Oesophagostomum*. PLoS Negl Trop Dis.

[12] Sripa B (2018). Tribendimidine: an alternative to praziquantel to treat human liver fluke infection?. Lancet Infect Dis.

[13] Pakharukova MY, Shilov AG, Pirozhkova DS, Katokhin AV, Mordvinov VA (2015). The first comprehensive study of praziquantel effects *in vivo* and *in vitro* on European liver fluke *Opisthorchis felineus* (Trematoda). Int J Antimicrob Agents.

[14] Mordvinov VA, Shilov AG, Pakharukova MY (2017). Anthelmintic activity of cytochrome P450 inhibitors miconazole and clotrimazole: *in vitro* effect on the liver fluke *Opisthorchis felineus*. Int J Antimicrob Agents.

[15] Ritz C, Baty F, Streibig JC, Gerhard D (2015). Dose-response analysis using R. PLoS One.

[16] Pakharukova MY, Pakharukov YV, Mordvinov VA (2018). Effects of miconazole/clotrimazole and praziquantel combinations against the liver fluke *Opisthorchis felineus in vivo* and *in vitro*. Parasitol Res.

[17] Xiao SH, Keiser J, Xue J, Tanner M, Morson G, Utzinger J (2009). Effect of single-dose oral artemether and tribendimidine on the tegument of adult *Clonorchis sinensis* in rats. Parasitol Res.

[18] Qian MB, Yap P, Yang YC, Liang H, Jiang ZH, Li W (2013). Efficacy and safety of tribendimidine against *Clonorchis sinensis*. Clin Infect Dis.

[19] Keiser J, Utzinger J, Xiao SH, Odermatt P, Tesana S (2008). *Opisthorchis viverrini*: efficacy and tegumental alterations following administration of tribendimidine *in vivo* and *in vitro*. Parasitol Res.

[20] Day TA, Botros S, Maule AG, Marks NJ (2006). Drug resistance in schistosomes. Parasitic flatworms: Molecular Biology, Biochemistry, Immunology and Physiology.

[21] Doenhoff MJ, Sabah AA, Fletcher C, Webbe G, Bain J (1987). Evidence for an immune-dependent action of praziquantel on *Schistosoma mansoni* in mice. Trans R Soc Trop Med Hyg.

[22] Thompson DP, Geary TG, Marr JJ, Nilsen TW, Komuniecki RW (2003). Helminth surfaces: structural, molecular and functional properties. Molecular Medical Parasitology.

[23] Keiser J, Vargas M (2010). Effect of artemether, artesunate, OZ78, praziquantel, and tribendimidine alone or in combination chemotherapy on the tegument of *Clonorchis sinensis*. Parasitol Int.

[24] Xiao SH, Xue J, Shen BG (2010). Tegumental alterations of adult *Schistosoma japonicum* harbored in mice treated with a single oral dose of mefloquine. Zhongguo Ji Sheng Chong Xue Yu Ji Sheng Chong Bing Za Zhi..

